# Tracking the evolution of serum antibody levels and influencing factors post-SARS-CoV-2 infection among community residents in Fuzhou City

**DOI:** 10.3389/fimmu.2025.1533102

**Published:** 2025-03-31

**Authors:** Xiaoyan Zheng, Qingquan Chen, Qiangbing Liao, Xiaoyang Zhang

**Affiliations:** ^1^ The Affiliated Fuzhou Center for Disease Control and Prevention of Fujian Medical University, Fuzhou, China; ^2^ The School of Public Health, Fujian Medical University, Fuzhou, China

**Keywords:** SARS-CoV-2, community residents, serum antibody, influencing factors, logistic regression

## Abstract

**Objective:**

To track the level of serum antibodies in Fuzhou residents and analyze the possible influencing factors of serum antibodies, so as to provide a scientific basis for the adjustment of population immunity and prevention and control strategies.

**Methods:**

Residents in the Fuzhou community who had symptoms of covid-19 infection or who had tested positive for nucleic acid or antigen since December 2022 were selected for the questionnaire survey and their sera were collected to analyze the trend of antibody changes, the antibody level was divided into high antibody group and low antibody group according to the literature data. The possible influencing factors of serum antibody level was analyzed by multivariate logistic regression model.

**Results:**

A total of 2,521 Fuzhou residents were adopted in the study, including 223 in the high antibody group and 194 in the low antibody group. A univariate analysis showed that, there were significant differences in age (Z=-4.028, P<0.00), occupation (χ^2^ = 18.591, P=0.005), typical symptoms after the first infection (χ^2^ = 9.784, P=0.002), history of surgery (χ^2^ = 29.542, P<0.001), symptoms lasting more than 2 weeks after the first infection (χ^2^ = 4.887, P=0.027), smoking (χ^2^ = 18.524, P<0.001) and drinking (χ^2^ = 19.578, P<0.001) between the high antibody group and the low antibody group. Multivariate regression models show that, age (OR= 1.011, 95%CI: 1.002~1.020, P=0.017), history of surgery (OR=4.956,95%CI: 2.606~9.423, P<0.001),smoking (OR=2.089, 95%CI: 1.002~4.355, P=0.049), drinking (OR=2.214, 95%CI: 1.066~4.600, P=0.033) were the risk factors affecting antibody level. Typical symptoms after the first infection (OR=0.224, 95%CI: 0.086~0.579, P=0.002) and symptoms lasting more than 2 weeks after the first infection (OR=0.432, 95%CI: 0.258~0.723, P=0.001) were protective factors. By observing the trend of antibody changes in 3, 6 and 9 months, we found that the level of IgG antibody showed a decreasing trend.

**Conclusions:**

The high level of protection was more likely to occur in young adults, people without operation history, people without smoking history, people without drinking history, people with typical symptoms after the first infection and symptoms lasting more than 2 weeks after the first infection. The level of IgG antibody was decreased in general, so it is necessary to strengthen immunization.

## Introduction

A global health emergency resulted from the severe acute respiratory syndrome coronavirus 2 (SARS-CoV-2), which caused the COVID-19 infection (1, 2). As of the end of December 2022, China has steadfastly implemented the “dynamic zero” COVID-19 policy, maintaining a relatively low level of domestic cases. However, following the adjustment of COVID-19 management to a Class B infectious disease on January 8, 2023, a significant surge in cases was observed.

To determine the main elements influencing the illness process and their long-term effects on immunity, it will be essential to comprehend the intricate immunological response triggered by SARS-CoV-2 infection. The span and potency of antibody responses provide valuable insight into the resilience of post-infection immunity (3).

Previous research on the dynamic changes of specific serum antibodies in COVID-19 patients predominantly focuses on the acute phase of the disease, with most studies analyzing antibody production and decay rates within the first few months of infection. This emphasis results in a significant gap in our understanding of long-term antibody dynamics, particularly regarding the persistence and protective efficacy of antibodies beyond the acute phase (4–6). Such a deficiency in the literature highlights the critical necessity for large-scale, longitudinal prospective studies. These studies are particularly needed in the aftermath of intense virus transmission episodes in specific regions, such as Fuzhou, where community-level immunity patterns may offer unique insights into the long-term impacts of the virus on population health.

Hence, this study involves 2,521 community residents from Fuzhou, employing questionnaires and collecting serum samples every three months to investigate the determinants and trends of serum antibody changes. By applying statistical methods to analyze factors influencing antibody levels and developing predictive models for high-risk groups, this research aims to provide a scientific basis for adjusting community immunity strategies and public health policies. This approach not only addresses a crucial gap in longitudinal immunological research post-high transmission but also enhances our preparedness for potential future outbreaks.

## Methods

### Participants

According to the formula of sample size, the reference at the end of 2022 the China provincial new virus infection monitoring data (7), take *p_1_* = 85%, the absolute maximum permissible error of the delta δ= 0.05, estimate the range of 80% - 90%, deff = 8.5. According to the formula, the effective sample size of our city was about 2000 people, and the loss rate of follow-up was expected to be 20%. The number of respondents was extended to 2400 people, and a total of 2521 community residents of Fuzhou were finally included as the research objects. Inclusion criteria for since December 2022, there have been SARS-CoV-2 infection symptoms or nucleic acid or antigen test is positive, different genders and all ages (≤18 years old, 18 to 59 years old, 60-79 years old, ≥80 years old) in a balanced distribution.

This prospective study employed multi-stage stratified random sampling. One street and one township were randomly selected from each county (city) district, and from each, 100 individuals were randomly chosen for a questionnaire survey and serological specimen collection. Thus, at least 200 people were surveyed in each county (city) district. These activities were conducted quarterly over a nine-month monitoring period on the community populations of the constituencies.

### Vaccination protocol

In Fuzhou City, the vaccination protocol comprised a three-dose regimen. The first dose served as the primary immunization, administered to individuals without prior SARS-CoV-2 infection. For those with confirmed prior infection, the initial dose was deferred until three months post-recovery to mitigate potential interference with immune response maturation. The second and third doses, designated as booster immunizations, were administered at intervals of three months following the primary dose and six months after the second dose, respectively. All vaccines utilized were inactivated SARS-CoV-2 formulations, produced by Sinopharm Beijing Institute of Biological Products Co., Ltd. (BBIBP-CorV) and Sinovac Biotech Ltd. (CoronaVac). These vaccines exhibited a documented shelf life of 24 months under standardized storage conditions.

### Questionnaire

In this study, the following information was collected by means of a questionnaire: gender, age, occupation, height, weight, whether there were typical symptoms after the initial infection, history of surgery, whether they had been vaccinated in the last three months, length of time to cure, whether they still had symptoms two weeks after the initial infection, history of drinking, history of smoking, and the presence of background diseases. The background diseases were referred to Underlying Medical Conditions Associated with Higher Risk for Severe COVID-19: Information for Healthcare Professionals published by the Centers for Disease Control, included diabetes mellitus, hypertension, autoimmune diseases, coronary atherosclerotic heart disease, tumors, and renal insufficiency, which were classified as underlying medical conditions.

### Laboratory tests

We collected venous blood samples in March, June, and September, using procoagulant tubes. Each sample, consisting of 3-5 ml of blood, was barcoded to correlate with its corresponding questionnaire. Samples were gently mixed, allowed to settle, and then centrifuged at 3000 rpm for 5-10 minutes. They were transported to the Centers for Disease Control within 24 hours. If not analyzed within 24 hours, samples were stored at 4°C. The serum IgG antibody levels were detected using the MAGLUMI IgG antibody detection kit based on chemiluminescence immunoassay. This involved centrifuging the samples at 2000 rpm for 10 minutes, labeling the antigen with a chemiluminescent agent, incubating for 20 minutes, and then performing the test on the machine. Results were exported to an Excel file for subsequent analysis.

### Statistical analysis

Based on serum antibody test values, subjects were categorized into high and low antibody level groups. Initially, a one-way analysis was performed. Measurement data were analyzed using either the t-test or the rank-sum test, while count data were evaluated with the chi-squared test. Significant variables identified through univariate screening were further analyzed to determine the influencing factors of serum antibody levels using a multifactorial logistic regression model. A two-sided p-value of less than 0.05 was considered statistically significant. All data were analyzed using *SPSS 26.0* software.

## Results

### Baseline

It was calculated that at least 2400 samples were needed, and ultimately, 2521 samples were collected. Literature review indicates that antibody levels ≥65 AU/mL are considered to represent high protection, while levels ≤20 AU/mL are considered low. Given that IgM appears earliest and decays fastest, it was excluded from the main analysis due to its large variability. After a significant infection wave in January 2023, antibody levels were naturally decaying by March; hence, the March IgG levels were analyzed to identify factors influencing serum antibody concentrations. From this analysis, 223 individuals were categorized into the high antibody group, and 194 into the low antibody group. In the high antibody group, the median age was 40 years (range: 15-65), and the median BMI was 22.27 (range: 19.60-24.97). Age did not follow a normal distribution (D=0.124, P<0.001), whereas BMI did (D=0.056, P=0.087). The group comprised 98 males (43.9%) and 125 females (56.1%). In the low antibody group, the median age was 57 years (range: 34.75-74), and the median BMI was 23.04 (range: 20.20-25.17), with both age and BMI not following a normal distribution (Age: D=0.091, P<0.001; BMI: D=0.095, P<0.001). This group included 93 males (47.9%) and 101 females (52.1%). In both groups, age and BMI were normally distributed as shown in **Table 1**. Information on the assignment of independent variables is shown in **Table 2**.

**Table 1:** Normality test of age and BMI.

	High antibody group		Low antibody group	
	Age	BMI	Age	BMI
Skewness	0.094	1.085	-0.398	1.322
Kurtosis	-1.256	5.529	-0.956	5.427
S-K value	0.124	0.056	0.091	0.095
P-value	<0.001	0.087	<0.001	<0.001

**Table 2:** Independent variable assignment information.

Variable		Assignment information
Group	X_1_	0=High antibody group, 1=Low antibody group
Gender	X_2_	1=Male, 2=Female
Occupations	X_3_	1= Retired persons, 2= Unemployed persons, 3= Farmers, 4= Students and children, 5= Cadres and staff, 6= Medical staff, 7= Others
Typical symptoms after initial infection	X_4_	1=Yes, 2=No
Background diseases	X_5_	1=Yes, 2=No
History of surgery	X_6_	1=Yes, 2=No
Vaccination history within the last 3 months	X_7_	1=Yes, 2=No
Length of time to cure	X_8_	1=within one week, 2=one to two weeks, 3=over two weeks
Symptoms of initial infection persisted for more than 2 weeks	X_9_	1=Yes, 2=No
Smoking	X_10_	1=Yes, 2=No
Drinking	X_11_	1=Yes, 2=No

### Univariate analysis of the influencing factors of serum antibody level

In the high antibody group and the low antibody group were significantly different in age (Z=-4.028,P<0.001), occupation (χ^2^ = 18.591, P=0.005), typical symptoms after the first infection (χ^2=^9.784, P=0.002), history of surgery (χ^2^ = 29.542, P<0.001), symptoms lasting more than 2 weeks after the first infection (χ^2^ = 4.887, P=0.027), smoking history (χ^2^ = 18.524, P<0.001), drinking history (χ^2^ = 19.578, P<0.001). There were significant differences in BMI (Z=-1.241, P=0.215), gender (χ^2^ = 0.666, P=0.414), background diseases (χ^2^ = 2.929, P=0.087), vaccination history in the last three months (χ^2^ = 0.312, P=0.576), length of cure (χ^2^ = 3.011, P=0.222), and the incidence of adverse reactions (Z=-1.241, P=0.215). P=0.222). The distribution of related factors and the analysis results are shown in **Table 3**.

**Table 3:** Distribution and analysis results of related factors in the two groups.

Variable		High antibody group (n=223)	Low antibody group (n=194)	Value of statistics	P-value
Age		40 (15, 65)	57 (34.75, 74)	Z=-4.028	<0.001
BMI		22.27 (19.60,24.97)	23.04 (20.20,25.17)	Z=-1.241	0.215
Gender	Male	98 (43.9%)	93 (47.9%)	χ^2^ = 0.666	0.414
	Female	125 (56.1%)	101 (52.1%)		
Occupation	Retired	24 (10.8%)	27 (13.9%)	χ^2^ = 18.591	0.005
	Unemployed	34 (15.2%)	35 (18.0%)		
	Farmers	7 (3.1%)	15 (7.7%)		
	Students and children	60 (26.9%)	31 (16.0%)		
	Cadres and staff	26 (11.7%)	15 (7.7%)		
	Medical staff	29 (13.0%)	16 (8.2%)		
	Others	43 (19.3%)	55 (28.4%)		
Typical symptoms after initial infection	Yes	216 (96.9%)	173 (89.2%)	χ^2^ = 9.784	0.002
	No	7 (3.1%)	21 (10.8%)		
Background diseases	Yes	63 (28.3%)	70 (36.1%)	χ^2^ = 2.929	0.087
	No	160 (71.7%)	124 (63.9%)		
History of surgery	Yes	18 (8.1%)	55 (28.4%)	χ^2^ = 29.542	<0.001
	No	205 (91.9%)	139 (71.6%)		
Vaccination history within the last 3 months	Yes	11 (4.9%)	12 (6.2%)	χ^2^ = 0.312	0.576
	No	212 (95.1%)	182 (93.8%)		
Length of time to cure	Within one week	105 (48.6%)	101 (57.4%)	χ^2^ = 3.011	0.222
	One to two weeks	70 (32.4%)	48 (27.3%)		
	Over two weeks	41 (19.0%)	27 (15.3%)		
Symptoms of initial infection persisted for more than 2 weeks	Yes	71 (31.8%)	43 (22.2%)	χ^2^ = 4.887	0.027
	No	152 (68.2%)	151 (77.8%)		
Smoking	Yes	15 (6.7%)	41 (21.1%)	χ^2^ = 18.524	<0.001
	No	208 (93.3%)	153 (78.9%)		
Drinking	Yes	15 (6.7%)	42 (21.6%)	χ^2^ = 19.578	<0.001
	No	208 (93.3%)	152 (78.4%)		

### Correlation analysis of antibody level (raw value)

IgG antibody level and age (P<0.001), whether there were typical symptoms after the first infection (P=0.041), surgical history (P<0.001), duration of cure (P=0.030), symptoms lasting more than 2 weeks after initial infection (P=0.005), smoking history (P<0.001), drinking history (P<0.001), but not with gender (P=0.576), BMI (P=0.342), occupation (P=0.788), vaccination history in the past three months (P=0.486) and underlying diseases (P=0.102). Among the variables related to antibody levels in this study, age (r=-0.194), typical symptoms after the first infection (r=-0.082), and symptoms lasting more than 2 weeks after the first infection (r=-0.114) were negatively correlated with antibody levels. History of surgery (r=0.182), length of cure (r=0.086), history of smoking (r=0.157), and history of drinking (r=0.174) were positively correlated with antibody levels, and the results are shown in **Table 4**.

**Table 4:** Correlation analysis of antibody levels (raw values).

	Correlation coefficient r	P-value
Age	-0.194	<0.001
BMI	-0.047	0.342
Gender	0.022	0.576
Occupation	0.010	0.788
Typical symptoms after initial infection	-0.082	0.041
Background disease	0.066	0.102
History of surgery	0.182	<0.001
Vaccination history within the last 3 months	-0.028	0.486
Length of time to cure	0.086	0.030
Symptoms of initial infection persisted for more than 2 weeks	-0.114	0.005
Smoking	0.157	<0.001
Drinking	0.174	<0.001

### Multivariate analysis of the influencing factors of serum antibody

Since occupation had statistical significance in the 2 test, but had no statistical significance in the correlation analysis; The length of cure time was not statistically significant in the 2 test, but was statistically significant in the correlation analysis. The results of the two tests were inconsistent, so it was not included in the multivariate regression analysis. Multivariate logistic regression analysis showed that age (P=0.017), typical symptoms after the first infection (P=0.002), symptoms after 2 weeks after the first infection (P=0.001), history of surgery (P&lt; 0.001), drinking history (P=0.033) and smoking history (P=0.049) were correlated with IgG antibody levels. Age (OR=1.011, 95%CI: 1.002-1.020, P=0.017), history of surgery (OR=4.956, 95%CI: 2.606-9.423, P&lt; 0.001), smoking history (OR=2.089,95%CI: 1.002-4.355, P=0.049) and drinking history (OR=2.214,95%CI: 1.066-4.600, P=0.033) were risk factors affecting antibody levels. Typical symptoms after the first infection (OR=0.224, 95%CI: 0.086-0.579, P=0.002) and symptoms lasting for more than 2 weeks after the first infection (OR=0.432, 95%CI: 0.258-0.723, P=0.001) were protective factors affecting the antibody level. The results are shown in **Table 5**.

**Table 5:** Logistic regression analysis.

Factor		β	SE	Wald χ^2^	OR	95%CI	P-value
Age		0.011	0.004	5.737	1.011	1.002-1.020	0.017
Typical symptoms after initial infection	No	–	–	–	–	1	–
	Yes	-1.498	0.486	9.519	0.224	0.086-0.579	0.002
History of surgery	No	–	–	–	–	1	–
	Yes	1.601	0.328	23.827	4.956	2.606-9.423	<0.001
Symptoms of initial infection persisted for more than 2 weeks	No	–	–	–	–	1	–
	Yes	-0.839	0.263	10.184	0.432	0.258-0.723	0.001
Smoking	No	–	–	–	–	1	–
	Yes	0.737	0.375	3.861	2.089	1.002-4.355	0.049
Drinking	No	–	–	–	–	1	–
	Yes	0.795	0.373	4.542	2.214	1.066-4.600	0.033
Constant		0.528	0.501	1.109	1.695		0.292

### Analysis of the trend of IgG antibody level

**Figure 1:**
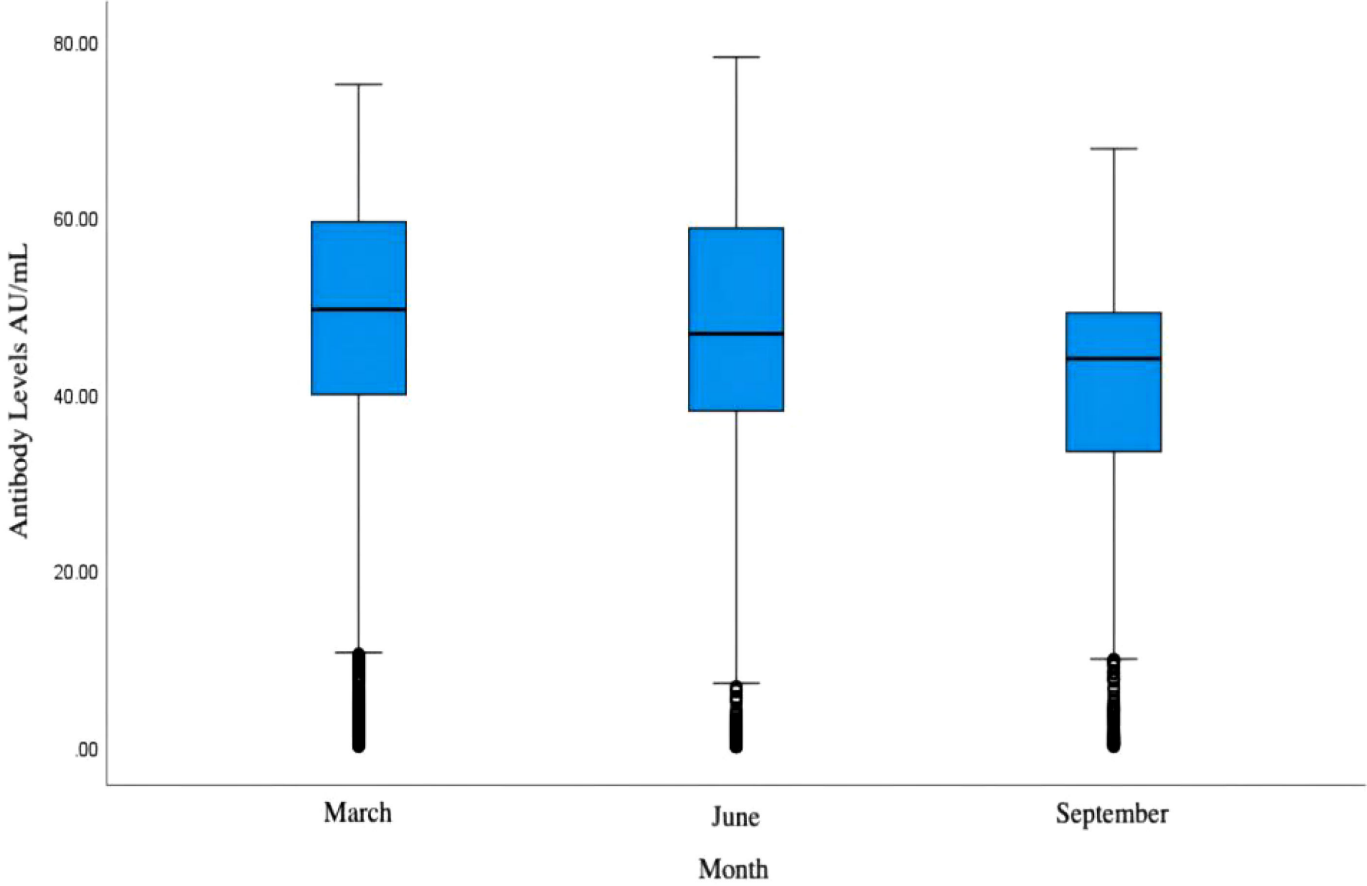
Overall IgG antibody levels of participants in March, June, and September.

Fuzhou experienced the first wave of infection peak from December 2022 to January 2023, and the antibody level had a natural decay process from January to March. After that, the second wave and the third wave of infection occurred in May and August of 2023. After one month, serum samples were collected again in June and September of this study. According to the test statistics, although the antibody level of some people increased slightly, the overall IgG antibody level in March, June and September still showed a downward trend, and the results are shown in **Figure 1**.

## Discussion

Although the community residents in Fuzhou experienced three infection peaks in January, May and August, the total IgG antibody level in March, June and September still showed a downward trend. According to the literature, the median time of specific IgG antibody seroconversion was 12 to 14 days after onset, rapidly increased and reached the peak in the third to fourth weeks, and decreased significantly 8 weeks after discharge (8).

Previous studies focused on antibody responses across different populations, time frames, and vaccination contexts. Our study reported a decline in IgG antibody levels in Fuzhou’s community after infection peaks, with demographic and clinical factors, such as age and smoking, influencing antibody levels. Protective factors included the presence of typical symptoms and prolonged symptoms post-infection. However, Wolszczak-Biedrzycka et al. (9) explored the effect of a third BNT162b2 dose on healthcare workers, showing higher antibody levels in previously infected individuals. This highlighted the booster’s role in enhancing immunity, in contrast to our study, which focused on natural immunity (9). This conclusion was strengthened by another study, showing a stronger antibody response in individuals who received the Pfizer/BioNTech booster compared to AstraZeneca, emphasizing vaccine-induced immunity over natural infection (10).In addition, one study examined the persistence of antibodies in healthcare workers 10 months post-vaccination, finding that prior infection leaded to more stable antibody levels (11). Prior infection and vaccination together resulted in stronger immunity (12). In conclusion, these findings all suggested the importance of considering individual vaccination history in immunity strategies.

Based on the results showing a downward trend in overall antibody levels, the newly produced immune antibodies are not sufficient to compensate for the declining antibody levels, suggesting that without the second and third waves of infection, antibody levels may have fallen more rapidly. Moreover, studies have shown that the duration of natural immunity is not long enough to resist the next variant (13). However, a moderate level of antibodies can protect people from severe damage caused by SARS-CoV-2, so it is necessary to strengthen immunization regularly to maintain the level of immunity (14).

The results of univariate analysis showed that gender, BMI, underlying disease, vaccination history in the past three months, and cure time had no significant effect on the level of antibody protection. The people with old age, history of surgery, smoking history, drinking history, no typical symptoms after the first infection, and symptoms did not last for more than 2 weeks after the first infection in the low antibody group were higher than those in the high antibody group. Although some studies have shown that compared with the vaccination of two doses of the inactivated SARS-CoV-2 vaccine, the vaccination of the third dose of the inactivated SARS-CoV-2 vaccine can significantly increase the levels of IgG antibodies and neutralizing antibodies and induce a higher level of immune protection (15, 16). However, due to the two years of COVID-19 epidemic, according to the national policy, the Chinese people have been generally vaccinated against COVID-19, and the number of people who have received booster shots is currently small. Moreover, the data collected in this study are from December 2022 onwards (because the body does not produce antibodies immediately after COVID-19 vaccine injection, and because of individual differences, Antibodies do not develop 100% of the time after vaccination.) “, while experiencing a large area of infection in January, which may account for the small effect of vaccination on antibody levels in this study.” In addition, some studies have shown that gender, BMI and underlying diseases can affect the antibody level (17–21), but in this study, there is no obvious correlation between gender, BMI and underlying diseases and the antibody level and change of community residents in Fuzhou, which may be confounded by other factors such as age. Whether gender, BMI and underlying diseases are the influencing factors of antibody level needs further detailed analysis.

Multivariate logistic regression model analysis showed that age, surgery history, drinking history, and smoking history were risk factors affecting antibody levels. Age is a risk factor, which may be related to lymphopenia, neutrophilia, inflammation and coagulation related indicators in elderly patients (≥65 years old). Increased neutrophils can lead to the formation of more significant neutrophil extracellular traps in the microvessels of critically ill patients (22, 23), which may be an enhancement factor in the pathogenesis of COVID-19 infection. These activated neutrophils and monocytes can accumulate in multiple organs (lung, heart, kidney, small intestine and liver, etc.), and increase the expression of related pro-inflammatory factors and inflammatory chemokines, thereby causing “cytokine storm” related inflammatory storm and aggravating pathological damage (24). In addition, the levels of IFN-γ and IL-2 in patients with acute COVID-19 (≥55 years old) are low, and the activation of T cells derived from dendritic cells is impaired, which will also lead to a reduced adaptive immune response (25). Surgical trauma has an impact on the immune system: studies have shown that the immunoglobulin level of patients decreases after surgery, and the degree of reduction is closely related to the level of surgical trauma (26). Long-term drinking, alcohol affects the function of adaptive immune response by acting on dendritic cells, T cells and other ways, and weakens the body’s immune response to pathogens on the whole (27). Moreover, alcohol has been shown to affect the signal of IFN and inhibit the body’s immune response to pathogens (28). Cigarette smoke can damage the maturation and function of dendritic cells, including the decreased ability to stimulate T cells and the inhibition of immune response (29). It can also reduce major histocompatibility class I antigen presentation and inhibit the immune response against virus-infected cells by inhibiting the activity of immunoproteasomes (30). Moreover, tobacco contains a variety of harmful chemicals such as nicotine, which can regulate innate and adaptive immune responses by interacting with acetylcholine receptors on the surface of immune cells (31).

In addition, the results of multivariate logistic regression model analysis showed that typical symptoms after the first infection and symptoms 2 weeks after the first infection were protective factors affecting the antibody level. This may be related to the severity of the disease, with typical symptoms after the first infection and symptoms lasting for more than two weeks indicating more severe disease. However, multiple relevant studies have shown that the level of IgG antibodies in patients with severe COVID-19 is higher, and the sustained high levels of specific IgM and IgG antibodies may be closely related to the severity of the disease (32, 33).

Finally, the inconsistency between the chi-square test and the correlation analysis of occupation and length of time to cure may be due to the loss of a part of the data information in the chi-square test analysis, which divided the antibody levels into high and low antibody groups.

This study also has certain limitations. First, the collected samples were randomly selected from community residents in Fuzhou. Although the sample size was large, there was loss to follow-up, incomplete data, and a certain degree of bias. Second, when analyzing the single factor, there were several confounding factors, and there was bias. Thirdly, although this study monitored the dynamic changes of antibody in March, June and September, the monitoring time was not enough. At the same time, only the data of IgG antibody in March were used to analyze the factors affecting serum antibody, which needs to be constantly summarized and improved in the follow-up related research.

## Conclusion

Age, history of surgery, smoking and drinking are risk factors for antibody levels. The presence of typical symptoms after the first infection and symptoms 2 weeks after the first infection were protective factors affecting antibody levels. The level of IgG antibody showed a downward trend. Therefore, it is necessary to strengthen immunization against COVID-19 infection regularly.

## Data Availability

The raw data supporting the conclusions of this article will be made available by the authors, without undue reservation.
